# Exploring the challenges and roles of nurses in delivering palliative care for cancer patients and co-morbidities in Ghana

**DOI:** 10.1186/s12904-023-01211-7

**Published:** 2023-08-28

**Authors:** Evans Osei Appiah, Awube Menlah, Jiayun Xu, Awuku Adomaa Susana, Boateng Susana Agyekum, Isabella Garti, Pascal Kob, Joyce Kumah

**Affiliations:** 1 Purdue University, West Lafayette, USA; 2 Charles Darwin University, Darwin City, Australia; 3 Purdue University School of Nursing, 502 University Street, West Lafayette, IN 47907-2069 USA; 4 Valley View University, Accra, Ghana; 5 Nursing Department, School of Nursing and Midwifery, Valley View University, Accra, Ghana; 6 Nursing Training College, Lawra, Upper West Region Ghana; 7 Ghana Christian University College, Accra, Ghana

**Keywords:** Nursing roles, Palliative care, Quality of life, Co-morbidities, Cancer

## Abstract

**Background:**

Patients suffering from chronic and life-threatening diseases receive inadequate palliative care in low-income countries, eventually leading to poor quality of life for these patients. Little is known about the experience of delivering palliative care in a low-resource country such as Ghana in comparison to higher-income countries. This study, therefore, aimed to assess the roles and challenges of nurses providing palliative care services for patients with cancer and life-limiting conditions at tertiary Hospitals in Ghana.

**Methods:**

Thirty oncology nurses at a tertiary Hospital in Ghana participated. All nurses were providing end-of-life care to patients with cancer. A qualitative exploratory-descriptive design and a semi-structured interview guide developed by the researchers were used. Interviews lasted on average forty minutes to 1 h were audio-recorded, and transcribed verbatim. Content analysis was carried out to generate themes and sub-themes.

**Findings:**

Participants were between the ages of 25 and 40 years. A higher percentage of females (*n* = 17, 57%) participated in the study than males (*n* = 13, 43%). Two main themes were generated which were the delivery of palliative care and the provision of home care services. The current roles of nurses were centered around pain management, home care services, spiritual needs, and psychological care. Challenges that hindered the implementation of palliative care included distress over expected and unexpected patient mortality, difficulty delivering bad news to patients and families, and frustration with health system resource shortages that negatively impacted patient care.

**Conclusion:**

Palliative care is one of the essential services provided for patients with life-limiting conditions, and nurses play an active role in the provision of this care. Further research is needed to determine the most effective ways to deliver this care, particularly in developing nations like Ghana.

**Supplementary Information:**

The online version contains supplementary material available at 10.1186/s12904-023-01211-7.

## Introduction

Palliative care aims at improving the quality of life for people with life-limiting conditions [1]. This includes managing the patient’s pain and symptoms and addressing the burden and psychosocial issues of both the patient and family [1, 2]. Other researchers have established that palliative care goes beyond the patient’s physical and psychological health to include the quality of life of the families of patients suffering from chronic disease in order to promote physical and psychological health [3–5].

Evidence suggests that patients suffering from chronic and life-limiting diseases such as prostate cancer and hepatocellular carcinoma receive inadequate care which could be linked to inadequate palliative care training for physicians and communication challenges [6, 7]. Similarly, poorly trained palliative care nurses were found to have limited palliative care knowledge and exhibited poor attitudes towards patients receiving palliative care than trained nurses [8, 9]. Some nurses possess the skills to effectively manage the symptoms of patients with chronic conditions despite the fact that their knowledge of palliative care may be lacking [10]. In Africa, various obstacles have been recognized as barriers to the successful implementation of palliative care, including late diagnosis, poverty, lack of knowledge, and limited access to palliative care services [11, 12].

Palliative care delivery is more prevalent in industrialized Western countries, such as the United States and the United Kingdom, than it is in African countries such as Ghana [13, 14]. In Ghana, there are only three hospitals that deliver palliative care services [14, 15]. These hospitals are the Korle-Bu Teaching Hospital, Tetteh Quarshie Memorial Hospital, and Komfo Anokye Teaching Hospital which are all located in urban areas. Thus, patients living in rural areas often face barriers to accessing palliative care services as the facilities and experts in this field are located in urban centers.

In Africa, including Ghana, there is a lack of understanding about the experiences of patients and nurses who provide palliative care services [16–18]. Understanding how palliative care can be successfully delivered and how it can be improved will benefit patients, their families, and the healthcare providers delivering this care. Therefore, the purpose of the study was to explore the roles and challenges of nurses providing palliative care services for patients with cancer and other co-morbidities at a tertiary hospital in Ghana which is one of the facilities providing palliative care.

### Research questions

1. What are the roles of nurses rendering palliative acre services to patients?
2. What challenges do nurses face whilst rendering palliative care services to patients with chronic conditions

## Methods

### Research design

A research design is a blueprint for connecting research questions and goals with relevant and viable empirical research methods [19]. A qualitative exploratory-descriptive design was employed to gain an understanding of palliative care nurses’ experiences, roles, and the difficulties they encountered at the palliative care unit. This approach was chosen as it is well-suited for investigating an area with limited prior research, such as palliative care in Ghana.

### Target population and sampling

The target population of this study was oncology nurses at a Teaching Hospital in Ghana one of the three palliative care centers in Ghana. The participants included in the study were oncology nurses who had worked for at least 2 years in the in the selected facility and had rendered care for patients receiving palliative care. Ten nurses were not included in the study as they reported they were unwilling to participate in the study after being contacted. Convenience sampling was used. Data saturation is the point at which qualitative research does not yield new information from additional data collection. It is often used as a criterion for determining sample size in qualitative research. In this study, recruitment was considered complete when. no new information was obtained from the interviews, indicating data saturation was reached. The sample size for this study was 30.

### Data collection tool

A semi-structured interview guide was developed by the researchers consisting of open-ended questions and probes to acquire in-depth information. It comprised three main sections: the socio-demographic section where the socio-demographic characteristics of the participants were asked while the other two sections probed the role of oncology nurses in palliative care and challenges faced by oncology nurses in the provision of palliative care, respectively.

### Procedure for data collection

Ethical clearance was obtained from the Dodowa Health Research Centre Institutional Review Board (DHRCIRB) with a reference number (DHRCIRB/106/10/21) and the medical director gave permission for this study to be conducted at the hospital. In addition to seeking consent from the study participants, the researcher also obtained permission from the nurse in charge. It was emphasized that participation in the study was voluntary and participants had the right to withdraw at any time.

After reading the informed consent form and seeking further clarification, the participants were given sufficient time to consider whether they wanted to take part in the study. Those who made a decision to partake in the study were made to sign the consent before the interview. During consent, questions on the interview guide were explained in simple terms to encourage understanding. The researchers listened attentively during the interview and reduced any distractions (e.g. turning off phones prior to the interview) that may have interrupted the participants as they expressed their knowledge and perceptions about palliative care. Furthermore, the researchers noted areas of interest that needed probing for further clarification. The participants were encouraged to further expand their ideas on the matter at hand. A voice recorder was used to record the interviews. The entire interview lasted from forty minutes to one hour. Data collection lasted for 2 months (from March 2022 to May 2022). The participants were thanked for their time at the end of each interview and they were reminded that they would be called upon for further clarification if needed.

### Piloting interview guide

A pilot interview was conducted at a private medical centre to test and revise the interview guide questions. Three participants who were oncology nurses, outside of the main study sample, were interviewed and provided feedback on the interview guide. The feedback was used to revise and further refine the semi-structured interview guide. The experiences of the three participants were not included in this analysis.

### Data analysis

Qualitative inductive content analysis was used for the data analysis. Inductive Content Analysis is primarily utilized in health research to analyze text-based data, such as written transcripts of verbal interactions or written documents [20, 21]. The goal of content analysis is to transform a large amount of data into a highly ordered summary of major findings, which entails examining raw data from verbatim-transcribed interviews to establish categories and themes. The open-coding process helps to identify patterns or themes in the data through the counting of frequencies, location of words, and phrases [22, 23]. The steps used for this content analysis were as follows:

*Familiarization*: The transcribed interviews were reviewed and re-read by the authors in order to gain an in-depth understanding of the transcripts. *Division of text into meaning units and condensing meaning units*: After the researchers familiarized themselves with the data, the text was subdivided based on the meaning of the information given. For example, all data about challenges and difficulties faced by nurses whilst providing care were grouped together. Short phrases or codes were used to summarize words in the transcripts whilst maintaining the meanings. Similar codes were collapsed together to form categories and sub-categories which were further grouped to form themes and subthemes.

Below is an illustration.

**Table:** 

Stages	Explanation	Examples
Familiarization	Reading and reading the verbatim transcripts	The authors repeatedly read the transcript to fully absorb the information obtained from the nurses. All the authors read the transcript
Condensation	Condensation is the act of simplifying and compressing a large amount of data into its most essential elements by means of methods such as summarization, synthesis and abstraction	The authors grouped similar information from each participant, then condensed the ideas into one sentence without changing their meaning For example, one of the condensed ideas was “Difficulties faced by oncology nurses whilst discharging their duties to patients”
Coding	Assigning labels or short descriptions	The ideas were condensed further into short labels of two to three words, while still keeping the meaning Example “Oncology nurses challenges”
Categorization and generation of themes	It is a method of organizing and understanding large amounts of information by recognizing patterns and commonalities	Similar codes were grouped together, and themes were identified for the similar codes to classify them For example all codes addressing difficulties faced by nurses were assigned a similar theme bellow “Challenges faced by oncology nurses”

Two major themes and 8 subthemes emerged

### Methodological rigor

Rigour is the process of ensuring the trustworthiness and reliability of a study’s findings [24]. There are four criteria: credibility, dependability, conformability, and transferability suggested for the assessment of rigor in qualitative research [25, 26]. This was to ensure that the results were reliable and robust [27]. See Table 1 for a detailed description of how trustworthiness was maintained in this study.

**Table 1:** Methodological rigor [17, 18]

Rigour criteria	Purpose	Application of rigour in our study
CREDIBILITY	Links study findings to reality to demonstrate the credibility of the research findings	1. Data collection lasted for 2 months (8 weeks) 2. The interview guide was pilot tested by 3 providers, and edited based on feedback prior to use 3. Three of the authors have palliative care backgrounds, and all are nurses who have cared for those with life-limiting illness
Dependability	Ensures results are consistent and repeatable	1. Inclusion and exclusion criteria, design, sampling, and data analysis procedures are detailed in the methods 2. Details on data collection processes were recorded in field notes
Confirmability	Ensures that a study result could be confirmed by other researchers [25, 26]	1. Researchers transcribed the interviews verbatim and also supported the results with verbatim quotes 2. Reflexive diaries were kept by the researcher outlining beliefs and experiences that may influence findings to ensure transparency 3. Triangulated the perspectives of various categories of nurses (Staff Nurses, Senior Staff Nurses, Nursing Officer, Senior Nursing Officer, and Principal Nursing Officer) during the design and analysis phases
Transferability	Extent to which study results may be used or transferred to other settings [27]	1. Data saturation was used to determine the sample size

## Results

### Sociodemographic characteristics of participants

A total of 30 nurses working in the oncology unit participated in the study. In all 40 nurses were contacted to partake in the study. Out of the 40 nurses contacted, 30 agreed to participate and none withdrew their consent. The socio-demographic characteristics analysed included: age, gender, religion, marital status, education level, rank, and the number of years the nurses had worked. As shown in Table 2, participants in the study were between the ages of 25 and 40. A higher percentage of females (*n* = 17, 57%) participated in the study than males (*n* = 13, 43%). Most of the participants were Christian (*n* = 26, 86%). Other details are found in Table 2.

**Table 2:** Socio-demographics of participants

Variable	Frequency (*n* = 30)	Percentage (%)
Age group		
25–30	9	30.00
31–40	21	70.00
Gender		
Male	13	43.00
Female	17	57.00
Religion		
Christian	26	86.00
Muslim	4	13.00
Marital status		
Single	7	23.33
Married	22	73.33
Divorced	1	3.333
Educational status		
Diploma	2	6.600
Bachelor	23	76.60
Masters	5	16.60
Rank		
(Staff Nurse)	8	26.66
(Senior Staff Nurse)	5	16.66
(Nursing Officer)	6	20.00
(Senior Nursing Officer)	11	36.66
(Principal Nursing Officer	0	0.000
Number of years working		
2–5 years	17	56.66
≥ 6 years	13	43.33

### Organization of themes

Two themes were established with eight sub-themes (Table 3). Pseudonyms were ascribed to each participant in each quote in order to maintain anonymity.

**Table 3:** Themes and subthemes

**1. Delivery of palliative care**	1. Pain management
	2. Provision of home care services to ensure continuity of care
	3. Providing spiritual care for coping and maintaining hope
	4. Psychological support for cancer patients
**2. Challenges faced by Oncology nurses**	1. Distress over expected and unexpected patient mortality
	2. Difficulty delivering bad news to patients and families
	3. Frustration with health system resource shortages

#### Theme 1: Delivery of palliative care

This theme encompasses the major roles oncology nurses took to care for their patients. Most of the nurses described different tasks they performed on a daily or weekly basis to support the patient’s quality of life. The common tasks were grouped into subthemes as detailed below.

### Pain management

Cancer patients often experience pain as a symptom due to tumors pressing on bones, nerves, and organs. Pain management is performed to alleviate discomfort. Some ways by which the pain were managed were recounted in the comments as follows:

“*Their pain is usually relieved with analgesics such as morphine and doreta (*an analgesic which is a combination of tramadol and paracetamol)*. The doctor prescribes these medications and they are given as at when the patient complains of pain. So, with palliative care we don’t say that patient will get addicted so let’s give the pain medications at this time or at that time, no, no, no. We try our maximum best to relieve them of their pain before they die.”* P4

“*Oh with their pain management, yes we give pain medications like doreta, morphine and ibuprofen but aside the medications, we also help them to assume a position they may feel comfortable in. I interact a lot with the patients, so when the pain fails to reduce even after drug administration, we encourage the person to lie in a way he or she prefers to reduce the pain. Some lie on their sides, some prefer holding their hands over the place they feel pain most.*” P8

Diversional therapy was an essential method noted by the study participants to distract the patient from the pain being felt.

*“Sometimes, recreational therapy is what we use to control their pain. For instance, you saw the fish at the outpatient department; it is kept there to serve as a source of recreational therapy purpose. So, when the patient is in pain and is sent there to watch the fishes swim, their minds are taken off the pain.”* P14

### Provision of home care services to ensure continuity of care

As a follow up, some of the nurses personally took it upon themselves to visit pay visits to patients at home to render care when patients had no one to assist them at the facility and/or patients could not care for themselves. Home care services were also provided upon request by some patients and family members who could afford such services. Some responses of the respondents were as follows.

*“There are times that patients will not be able to come especially when the person has no one to assist him or her to come to the facility. We have nurses among us who decide to visit patients at home to assist them with personal hygiene and other needs ”* P15

*“At times, some nurses volunteer to visit patients at home to dress wounds and monitor medication compliance when patients are unable to come to the facility, promoting comfort. .”* P30

Home care services were also provided to patients who preferred to be cared for at home and could afford the services.

*“Hmm for the home care services, based on what I have experienced over the years, the home care is not just for patients who are very weak and cannot come to the hospital. Some patients prefer being treated at home other than traveling long hours This is mostly requested by patients who are well-to-do and could pay for the services.”* P24

### Providing spiritual care for coping and maintaining hope

Spiritual care was a very important factor in assuring the overall well-being of palliative care patients. Ensuring the provision of spiritual care helped patients improve their coping skills and increased feelings of hope, which led to decreased anxiety of the patients. The views of some respondents on the spiritual needs of patients were as follows.

*“We assist patients in meeting their spiritual needs by praying with them or inviting their spiritual leaders to do so, depending on their beliefs. This has been found to alleviate anxiety for some patients” P28*

*“Spiritual care is necessary for the sense that sometimes most of the patients think that their condition is being manipulated by external forces so with spiritual care, they know that those forces are under control and come to accept the reality of their condition. ”* P29

Nurses recognized the value of spiritual care for patients and saw this as a critical component to patient’s having positive moods.

*“For spiritual care, we see it to be very crucial for patients receiving palliative care. This is why we have a man of God here at the radiotherapy unit who comes here every morning to speak to them before the clinical psychologist comes in this has helped most patients here to feel less anxious.”* P1

*"Patients benefit from spiritual care, particularly those who feel guilty about their suffering. It helps them understand it's not their fault, makes peace with their past, and forgives themselves, leading to feelings of hope and happiness”P 11.*

### Psychological support for cancer patients by nurses, psychologists, and relatives

Many cancer patients endure some form of psychological distress, and the nurses, along with the patient's family members, offer psychological support to patients with such illness. The nurses reported that this support is vital in reducing patient anxiety and assisting them in dealing with the grieving process without any issues.

“*We have a psychologist here in our unit who speaks with the patients every day before treatment begins. This helps the patient to know how to cope with the disease condition. Even though we have psychologist here to speak to the patients, we as oncology nurses also play our part by letting them know how their condition came about, the stage it is, and what we can do to help.*.” P5

“*We provide psychological support by paying attention to patients and relatives as they speak, showing empathy, encouraging them and making them see that they are not alone. It makes them feel like someone cares and makes them more hopeful”* P18

Other participants also indicated that psychological care for such patients could be provided by the patients’ relatives. Nurses facilitated the engagement of relatives in patient care by encouraging this behaviour while caring for patients.

*“Patient’s relatives also have a role to play too. In providing psychological care, their choice of words is very crucial. In the sense that what they tell the patient can them recover early or deteriorate their condition. So it is our duty to engage them in the care”* P16

#### Theme 2: Challenges faced by oncology nurses

The current study revealed that the nurses caring for patients who need palliative care also faced challenges that needs to be addressed in order to enhance their care delivery. The challenges described by the nurses included expected and unexpected patient mortality, difficulty delivering bad news to patients and family, and frustration with health system resource shortages that negatively impacted patient care.

### Distress over expected and unexpected patient mortality

Patient mortality is a significant concern for healthcare professionals and can cause distress for those who are expected to pass away as well as for those who pass away unexpectedly. Below are quotes to depict that some nurses were worried and frustrated following patients death.

*“It becomes a challenge for us the nurses because even though death is expected, some patients look a little healthier and we expect them to live longer so their death is unexpected and it becomes a shock to us the nurses considering the efforts we put into their care”* P20

*“A patient once died here and after thorough investigation we realized that they were combining the treatment we were given with herbal treatment without our notice which led to a sudden change in her condition and her sudden dismissal. It is a worry when such incidence happens under our care but it is patient right so we cannot do anything about that.* P9

*“We once admitted a patient with cancer who was brought in when the cancer has spread to the whole breast and was discharging. The patient had lost weight and was in pain. The patient said that their relatives took him to see an herbalist for 3 months before coming to the hospital. The patient died within 30 minutes of starting chemotherapy. This was disturbing because if they have come early, she would no have gone through that pain with the discharges prior to her departure.”* P22

### Difficulty delivering bad news to patients and families

Another challenge nurses experienced was difficulty breaking the news of the patient’s death to their family members and managing the family’s reactions thereafter. Even if nurses felt skilled in delivering bad news, they were not well prepared to manage the family’s emotions and know how to provide support. These experiences were especially pronounced when the patient was younger. Instances of such occasions were described below.

**“***Some family members even though know that their relatives are near death and we are just managing their symptoms to improve their quality of life still hope that their relatives will stay a bit longer so it becomes difficult when they bring their relatives and the next moment, they are told the person could not make it. It is a challenge for us to break such news especially when they least expected it. Even though we try our best to comfort them, hmm … it is still not easy because of how relatives ”* P10

*A 35 years old woman (patient) reported to the hospital with her mother some time ago. After the assessment, she had cervical cancer, which had spread to the lungs. It was difficult to break this news to the patient and her mother considering the age of the patient. We had to break the news anyway after counseling services were organized for the patient and her mother”* P26

“*Breaking bad news is not of a challenge to me because I am an expert in that because I have done it for so many years and I am always called upon to do that if I’m on duty, but my only challenge is how their reaction will be following the news”* P13

### Frustration with health system resource shortages that negatively impacted patient care

Lack of adequate resources and resource personnel was a major concern. This included limited rooms for treatment, inadequate palliative care nursesand an absence of some resources for radiation therapy. Most expressed frustration over the amount of time patients needed to wait before getting chemotherapy and the lack of control they had over these resource gaps. These challenges are illustrated by the quotes below.

*“One main challenge is that we have limited rooms for radiotherapy and chemotherapy treatment. Because here is a referral center, our rooms for these basic treatments should have been bigger. We have only four rooms for these treatments and the sizes are too small. The chemotherapy unit can only occupy at most 5 patients at a time and this slow down the delivery of care as just a few patients are being taken care of and the rest are rescheduled for another day. Some patient relatives respond in anger and we have to calm them down.”* P27

*“The doctors here are few compared to other units. Sometimes patients will be in pain for a long before we get a doctor to attend to them and it makes us emotional distress to see patients in pain. When this happens, we make a phone call to any doctor available to attend to them. and most of the nurses here have learned on the job, we are not experts in palliative care.”* P13

*“The nurses working at the oncology units are general nurses and few are oncology nurses but we do not have nurses who have been trained as palliative care nurses, this is because it is recently that Ghana college have introduced palliative nursing, most of the skills and knowledge we have was acquired on the field as we practice. P11*

*“There are situations where patients come and there is no bed, the patient will have to be rescheduled. Imagine a terminally ill patient who is on dialysis at the renal unit who needs this service at least 3 times a week to survive being rescheduled because of no bed syndrome. This has a negative impact on the quality of care the patient is expected to get and causes us to worry.”* P3

## Discussion

This study explored the experiences of nurses who were working on an oncology unit in a tertiary hospital in Ghana. The findings revealed the palliative care approach performed by Ghanaian nurses and the challenges they faced when providing palliative care.

A key discovery was that nurses reported on the typical pain medications they prescribe to patients and their effectiveness in providing relief while avoiding addiction and maintaining patients' quality of life. Common analgesics which were prescribed and administered when patients had pain included Morphine (Avinza), Doreta (Doxpen), and Ibuprofen (Nurofen, Brufen, Calprofen). This finding corresponds with WHO guidelines for cancer pain management and other other studies that indicate that pain was best controlled with analgesics such as morphine [28, 29]. Effectiveness of the World Health Organization cancer pain relief guideline 2016 As with previous studies, pain management also included the use of non-pharmacologic practices such as patient positioning for comfort, relaxation exercises, and massage [30, 31]. The focus on pain was not surprising given that pain management is considered a major pillar in palliative care [31, 32] and is a major troublesome symptom among patients with metastasized cancers [33].

The study uncovered an unexpected finding, in which some of the nurses were voluntarily providing home-based care to patients who were no longer able to receive end-of-life care in the hospital. The services offered by these nurses include wound care, and bathing, which were given to patients in the comfort of their own homes. This indicates that the nurses in the study were extending their care beyond the hospital setting to provide more personalized and compassionate care to patients during the end-of-life stage. Furthermore, it indicates that they recognized the preference of many patients to receive care in their own homes rather than in a hospital. The nurses in this study went above and beyond their usual duties to ensure that their patients received the best possible care. Hospital nurses typically only provide care to patients in the hospital, and in Ghana, Patients who require home care usually have to engage private homecare nurses. Therefore, it was surprising that some of these nurses have to volunteer to provide care to patients who could not afford treatment in their homes. On the contrary, Salifu et al. study revealed that family caregivers in Ghana sometimes provided palliative and end-of-life care unsupported by health staff due to reasons such as lack of adequate staff to do home visit, lack of money to pay for such private services [7]. The current study finding was also inconsistent with another study that reported inadequate staff support for patients receiving end-of-life care [34]. Home-based end-of-life care is beneficial as found in several studies since it can help increase adherence to treatment, help in early detection of worsening illness, lessen symptoms, and improve caregiver satisfaction [35, 36]. Similarly, a study ascertained that palliative care services at home improve patient satisfaction, and use of palliative care services by patients, and prevented unnecessary expenses [37]. The current study also revealed that some patients with higher socioeconomic status requested nursing services in their homes. This discovery implies that access to home-based care for end-of-life patients may be influenced by factors such as wealth and power. It is likely that those who are financially well-off and have more influence may be able to request and receive these services, while those who are less well-off may not have the same access. This creates a disparity in the quality of life for patients with similar conditions, based on their socioeconomic status, which is a cause for concern. This finding raises concerns, especially in Ghana where home palliative care services are typically only available in urban areas. Thus, there are care disparities in which patients with low socio-economic status in the urban areas may have access to home palliative care services and/or not be able to afford palliative care home services. It is therefore imperative that governmental and non-governmental support are provided for patients who would benefit from palliative care services in the home.

The nurses in this study made sure that the spiritual well-being of their patients was not overlooked in their care. By addressing the spiritual aspect, they aimed to provide a comprehensive and holistic approach to care, which includes providing hope, comfort and an improved quality of life for patients. This finding aligns with the results of previous studies that emphasized the significance of spiritual care as an essential aspect of palliative care. It was found that spiritual care can provide a sense of serenity, being present, enabling and alleviating patients' suffering [38, 39]. The World Health Organization (WHO) recommends that spiritual care be incorporated as a vital aspect of palliative care for patients, as it can aid in reducing their anxieties and provide comfort [29, 40]. Based on these reasons, spiritual care workshops to effectively provide spiritual care may be beneficial in educating health care providers who provide palliative care services to terminally ill patients [41].

The current study also revealed that the nurses experienced emotional distress, psychological trauma, and frustrations as a result of following death of some of their patients due to the relationship that exists between them. Even though palliative care neither aims to hasten death nor prolong life, it was not surprising the nurses were frustrated over the increase in mortalities due to the energy, time spent, and the relationships formed with these patients. Similarly, evidence suggests that due to the increased mortalities of patients, some nurses were feeling sad and helpless [42].

It was reported that nurses (even the few who felt comfortable breaking bad news) were grieved by the events that unfolded when bad news (e.g. the cervical cancer diagnosis, etc.) was delivered to patients and relatives, and the aftermath of the bad news delivery. This finding is consistent with studies that indicated that delivering bad news to patients and relatives was one of the most difficult tasks nurses encounter as it might alter patients’ views about themselves [43–45]. Hence a multidisciplinary approach (psychologist, chaplains, social workers, and family involvement) is needed to render holistic care to patients some patients who are on admission [46] A multi-disciplinary approach may be helpful in breaking bad news to patients and relatives, especially among providers who do not feel comfortable or are not experienced with having difficult conversations [47, 48]. There is therefore the need for special training courses to help healthcare providers improve their knowledge and skills in delivering bad news and supporting patients/relatives after the delivery is complete [49].

A lack of adequate resources and resource personnel were major challenges faced by oncology nurses in Ghana. These resource gaps were beyond the control of nursing staff and caused them to be unable to provide quality palliative care. In Ghana, only a few providers are trained in palliative care and only three (Korle Bu Teaching Hospital (KBTH), the Tetteh Quarshie Memorial Hospital, and the Komfo Anokye Teaching Hospital (KATH)) hospitals provide palliative care [14, 15, 17]. The palliative care infrastructure in Ghana is very limited and primarily concentrated in the urban areas. Even in industrialized countries, resource issues such as lack of care continuity and scheduling are obstacles to palliative care delivery [50]. It is therefore imperative that more attention is directed toward palliative care resources by the government, policymakers, non-governmental organizations, and other stakeholders. Having enough resources to deliver quality and timely palliative care will help improve patient quality of life and reduce burnout and increase satisfaction among palliative care nurses [51, 52].

## Conclusion

Palliative care is crucial for individuals with terminal illnesses, and nurses are actively involved in providing this care. Our sample of Ghanaian oncology nurses were found to perform the typical tasks associated with palliative care practitioners, such as managing pain and providing spiritual support, despite facing significant obstacles that were beyond their control, such as limited resources and patients and families utilizing non-conventional herbal treatments. This study provides insight into how palliative care is delivered in low-resource settings in a developing country. More research is needed to determine how to improve and expand palliative care delivery, especially in developing countries such as Ghana.

### Supplementary Information

**Additional file 1.** Interview guide.

## Data Availability

All data generated or analyzed during this study will be made available upon request from the corresponding author.

## Abbreviations

KATH: Komfo Anokye Teaching Hospital
WHO: World Health Organization
KBTH: Korle-Bu Teaching Hospital
DHRCIRB: Dodowa Health Research Centre Institutional Review Board

## References

[1] Kelley AS, Morrison RS (2015). Palliative care for the seriously ill. N Engl J Med.

[2] Ullrich A, Ascherfeld L, Marx G, Bokemeyer C, Bergelt C, Oechsle K (2017). Quality of life, psychological burden, needs, and satisfaction during specialized inpatient palliative care in family caregivers of advanced cancer patients. BMC Palliat Care.

[3] Aparicio M, Centeno C, Carrasco JM, Barbosa A, Arantzamendi M (2017). What are families most grateful for after receiving palliative care? Content analysis of written documents received: a chance to improve the quality of care. BMC Palliat Care.

[4] Akard TF, Hendricks-Ferguson VL, Gilmer MJ (2019). Pediatric palliative care nursing. Ann Palliat Med.

[5] Roth AR, Canedo A, Balabanova R, Chauhan V (2020). My life, My way: a quality improvement Program to increase advance care planning in a Racially and Ethnically Diverse patient Population. J Pain Symptom Manage.

[6] Laube R, Sabih AH, Strasser SI, Lim L, Cigolini M, Liu K (2021). Palliative care in hepatocellular carcinoma. J Gastroenterol Hepatol.

[7] Salifu Y, Almack K, Caswell G (2021). ‘My wife is my doctor at home’: a qualitative study exploring the challenges of home-based palliative care in a resource-poor setting. Palliat Med.

[8] Suwanabol PA, Reichstein AC, Suzer-Gurtekin ZT, Forman J, Silveira MJ, Mody L, Morris AM (2018). Surgeons’ perceived barriers to palliative and end-of-life care: a mixed methods study of a surgical society. J Palliat Med.

[9] Gedefaw G, Wondmieneh A, Getie A, Bimerew M, Demis A (2021). Estimating the prevalence and risk factors of obstetric fistula in Ethiopia: results from demographic and health survey. Int J Women's Health.

[10] Sayed AE, Atef Ibrahiem Elzehiri D, Hassan Mohamady S (2021). Effect of Palliative Care Training Package on Nurses’ Performance regarding Gynecologic Cancer. Egyptian Journal of Health Care..

[11] Kim S, Lee K, Kim S (2020). Knowledge, attitude, confidence, and educational needs of palliative care in nurses caring for non-cancer patients: a cross-sectional, descriptive study. BMC Palliat Care.

[12] LaVigne AW, Gaolebale B, Maifale-Mburu G, Grover S (2018). Palliative care in Botswana: current state and challenges to further development. Ann Palliat Med.

[13] Adejoh SO, Boele F, Akeju D, Dandadzi A, Nabirye E, Namisango E, Namukwaya E, Ebenso B, Nkhoma K, Allsop MJ (2021). The role, impact, and support of informal caregivers in the delivery of palliative care for patients with advanced cancer: a multi-country qualitative study. Palliat Med.

[14] Agom DA, Onyeka TC, Iheanacho PN, Ominyi J (2021). Barriers to the provision and utilization of palliative care in Africa: a rapid scoping review. Indian J Palliat Care.

[15] George N, Bowman J, Aaronson E, Ouchi K (2020). Past, present, and future of palliative care in emergency medicine in the USA. Acute medicine & surgery.

[16] Gadoud A, Kane E, Oliver SE, Johnson MJ, Macleod U, Allgar V. Palliative care for non-cancer conditions in primary care: a time trend analysis in the UK (2009-2014). BMJ Support Palliat Care. 2020:bmjspcare-2019-001833. 10.1136/bmjspcare-2019-001833. 10.1136/bmjspcare-2019-001833 31932476

[17] Ofosu-Poku R, Owusu-Ansah M, Antwi J. Referral of Patients with Nonmalignant Chronic Diseases to Specialist Palliative Care: A Study in a Teaching Hospital in Ghana. Int J Chronic Dis. 2020;2020:8432956. 10.1155/2020/8432956. 10.1155/2020/8432956 PMC7102405 32258093

[18] Anyane G, Ofosu-Poku R, Delali Dzaka A. Patients’ Perceived Outcome of Specialist Palliative Care at the Komfo Anokye Teaching Hospital, Ghana. 2021;4(1):284–90.

[19] Van Wyk B. Research design and methods Part I. University of Western Cape. 2012.

[20] Vears DF, Gillam L (2022). Inductive content analysis: a guide for beginning qualitative researchers focus on health professional education. Multi Discip J.

[21] Karanikola MN. Content analysis in critical and emergency care: a discussion paper. connect. World Critic Nurs. 2019;13(1).

[22] Brysiewicz EC (2017). A hands-on guide to doing content analysis. Afr J Emerg Med.

[23] Miles MB, Huberman AM. Qualitative data analysis: an expanded sourcebook. Sage; 1994.

[24] Hamberg K, Johansson E, Lindgren G, Westman G (1994). Scientific rigour in qualitative research—examples from a study of women’s health in family practice. Fam Pract.

[25] Ćwiklicki M, Urbaniak A (2018). Methodological rigour in descriptions of case-study research in polish academic articles on management. Przedsiębiorczość Zarządzanie..

[26] Ryan-Nicholls KD, Will CI. Rigour in qualitative research: mechanisms for control. Nurse Res. 2009;16(3):70–85. 10.7748/nr2009.04.16.3.70.c6947. 10.7748/nr2009.04.16.3.70.c6947 19425402

[27] Forero R, Nahidi S, De Costa J, Mohsin M, Fitzgerald G, Gibson N, McCarthy S, Aboagye-Sarfo P (2018). Application of four-dimension criteria to assess rigour of qualitative research in emergency medicine. BMC Health Serv Res.

[28] Ho JF, Yaakup H, Low GS, Wong SL, Tho LM, Tan SB (2020). Morphine use for cancer pain: A strong analgesic used only at the end of life? A qualitative study on attitudes and perceptions of morphine in patients with advanced cancer and their caregivers. Palliat Med.

[29] Carlson CL (2016). Effectiveness of the World Health Organization cancer pain relief guidelines: an integrative review. J Pain Res.

[30] Hilfiker R, Meichtry A, Eicher M, Balfe LN, Knols RH, Verra ML, Taeymans J (2018). Exercise and other non-pharmaceutical interventions for cancer-related fatigue in patients during or after cancer treatment: a systematic review incorporating an indirect-comparisons meta-analysis. Br J Sports Med.

[31] Lopes-Júnior LC, Rosa GS, Pessanha RM, Schuab SIPC, Nunes KZ, Amorim MHC. Efficacy of the complementary therapies in the management of cancer pain in palliative care: a systematic review. Rev Lat Am Enfermagem. 2020;28:e3377. 10.1590/1518-8345.4213.3377. 10.1590/1518-8345.4213.3377 PMC7529450 33027406

[32] Krakauer EL, Kwete X, Verguet S, Arreola-Ornelas H, Bhadelia A, Mendez O, Ali Z, Allende S, Cleary JF, Connor S, Danforth K (2017). Palliative care and pain control. Disease control priorities: improving health and reducing poverty.

[33] Morris AT, Gabert-Quillen C, Friebert S, Carst N, Delahanty DL (2016). The indirect effect of positive parenting on the relationship between parent and sibling bereavement outcomes after the death of a child. J Pain Symptom Manage.

[34] Bayuo J, Kyei Baffour P. Healthcare Staff Perceptions Regarding Barriers and Enablers to End-of-Life Care Provision in Non-Palliative Care Settings in Ghana: A Multicentre Qualitative Study. Omega (Westport). 2022:302228211072970. 10.1177/00302228211072970. 10.1177/00302228211072970 35090354

[35] Gomes B, Calanzani N, Curiale V, McCrone P, Higginson I. J.Effectiveness and cost-effectiveness of home palliative care services for adults with advanced illness and their caregivers. Cochrane Database Syst Rev. 2013(6). 10.1002/14651858.CD007760.pub2 PMC4473359 23744578

[36] Fernandes R, Braun KL, Ozawa J, Compton M, Guzman C, Somogyi-Zalud E (2010). Home-based palliative care services for underserved populations. J Palliat Med.

[37] Labson MC, Sacco MM, Weissman DE, Gornet B, Stuart FB (2013). Innovative models of home-based palliative care. Optimizing home health care: enhanced value and improved outcomes.

[38] Gijsberts MJ, Liefbroer AI, Otten R, Olsman E (2019). Spiritual care in palliative care: a systematic review of the recent European literature. Medical Sciences.

[39] Mahilall R, Swartz L (2021). Spiritual care practices in hospices in the Western cape, South Africa: the challenge of diversity. BMC Palliat Care.

[40] Sepúlveda C, Marlin A, Yoshida T, Ullrich A (2002). Palliative care: the World Health Organization's global perspective. J Pain Symptom Manage.

[41] Karimi R, Mousavizadeh R, Mohammadirizi S, Bahrami M (2022). The effect of a spiritual care program on the self-esteem of patients with cancer: a quasi-experimental study. Iran J Nurs Midwifery Res.

[42] Kostka AM, Borodzicz A, Krzemińska SA (2021). Feelings and emotions of nurses related to dying and death of patients–a pilot study. Psychol Res Behav Manag.

[43] Malloy P, Virani R, Kelly K, Munévar C (2010). Beyond bad news: communication skills of nurses in palliative care. J Hosp Palliat Nurs.

[44] Fallowfield L, Jenkins V (2004). Communicating sad, bad, and difficult news in medicine. Lancet.

[45] Dias L, Chabner BA, Lynch TJ, Penson RT (2003). Breaking bad news: a patient’s perspective. Oncologist.

[46] Swami M, Case AA. Effective palliative care: what is involved? Oncology (08909091). 2018;32(4). 29684230

[47] Spiegel W, Zidek T, Maier M, Vutuc C, Isak K, Karlic H, Micksche M (2009). Breaking bad news to cancer patients: survey and analysis. Psychooncology.

[48] Alshami A, Douedi S, Avila-Ariyoshi A, Alazzawi M, Patel S, Einav S, Surani S, Varon J (2020). Breaking bad news, a pertinent yet still an overlooked skill: an international survey study. Healthcare.

[49] Jeraine TI, Wakefield A. The clinical effectiveness of breaking bad news educational programme for registered nurses: a review of the recommendations. Singapore Nurs J. 2018;45(2).

[50] Midtbust MH, Alnes RE, Gjengedal E, Lykkeslet E (2018). Perceived barriers and facilitators in providing palliative care for people with severe dementia: the healthcare professionals’ experiences. BMC Health Serv Res.

[51] Abu-Odah H, Mikati D, Arawi T (2021). Deconstructing palliative care in areas of armed conflict: needs, challenges, and concerns. Handbook of healthcare in the Arab World.

[52] World Health Organization. Quality health services and palliative care: practical approaches and resources to support policy, strategy and practice. https://www.who.int/publications/i/item/9789240035164.

