# Supplementary material for: Exploring the challenges and roles of nurses in delivering palliative care for cancer patients and co-morbidities in Ghana
Source: BMC Palliat Care. 2023 Aug 28;22:121. doi: 10.1186/s12904-023-01211-7 (PMC10464455; doi:10.1186/s12904-023-01211-7)
Supplement: Supplementary file 1 — Additional file 1. Interview guide. [file 12904_2023_1211_MOESM1_ESM.docx]

# APPENDIX

**INTERVIEW GUIDE**

**Dear Respondent,**

Our research focuses on investigating the challenges and roles of nurses involved in providing palliative care for cancer patients with co-morbidities in Ghana. We have chosen you as one of the participants for this study. Your valuable insights will be used solely for research purposes. If you agree to participate, we kindly request a 45-60 minute interview to discuss aspects such as your background, responsibilities, and the difficulties encountered in delivering palliative care. Please be assured that all collected data will be treated with strict confidentiality. Your anonymity is guaranteed, and there is no need to provide any identifying information. **Section A: Demographic data**

Tell me about yourself.

Probe

- Age
- Religion
- Educational level
- Marital status
- Working experience
- Rank

**Section B:** **Roles of Nurses in Delivering Palliative Care for Cancer Patients**

**Probes**

1. What are your roles as a palliative care nurse?
2. How do you provide emotional and psychological support to patients with cancer?
3. Can you discuss the challenges you face in managing pain and symptom control for patients receiving palliative care?

Probes

Your day-to-day activities

Pain management

Emotional support

**Section C: Challenges Faced by Nurses Delivering Palliative Care for Cancer Patients**

1. What challenges do you face daily?
2. Why do you face those challenges?
3. How do you address these challenges?
4. Are there any resource-related challenges?
5. How do you handle the high workload and time constraints in providing comprehensive care to oncology patients?
